# Simultaneous Quantification of Propylthiouracil and Its *N*-β-d Glucuronide by HPLC-MS/MS: Application to a Metabolic Study

**DOI:** 10.3390/ph14111194

**Published:** 2021-11-20

**Authors:** Min Li, Qingfeng He, Li Yao, Xiaofeng Wang, Zhijia Tang, Xiao Zhu, Hai-Shu Lin, Xiaoqiang Xiang

**Affiliations:** 1 Department of Clinical Pharmacy and Pharmacy Administration, School of Pharmacy, Fudan University, Shanghai 201203, China; 19211030067@fudan.edu.cn (M.L.); qf_he@fudan.edu.cn (Q.H.); 19261030004@fudan.edu.cn (L.Y.); 20211030100@fudan.edu.cn (X.W.); zjtang@fudan.edu.cn (Z.T.); xiaozhu@fudan.edu.cn (X.Z.); 2 College of Pharmacy, Shenzhen Technology University, Shenzhen 518118, China

**Keywords:** propylthiouracil, propylthiouracil glucuronide, in vitro, human liver microsomes, UGT1A9, HPLC-MS/MS

## Abstract

Propylthiouracil (PTU) is commonly prescribed for the management of hyperthyroidism and thyrotoxicosis. Although the exact mechanism of action is not fully understood, PTU is associated with hepatoxicity in pediatric population. Glucuronidation mediated by uridine 5′-diphospho-glucuronosyltransferases (UGTs), which possess age-dependent expression, has been proposed as an important metabolic pathway of PTU. To further examine the metabolism of PTU, a reliable HPLC-MS/MS method for the simultaneous quantification of PTU and its N-β-D glucuronide (PTU-GLU) was developed and validated. The chromatographic separation was achieved on a ZORBAX Extend-C18 column (2.1 × 50 mm, 1.8 μm) through gradient delivery of a mixture of formic acid, methanol and acetonitrile. The electrospray ionization (ESI) was operated in its negative ion mode while PTU and PTU-GLU were detected by multiple reaction monitoring (MRM). This analytical method displayed excellent linearity, sensitivity, accuracy, precision, recovery and stability while its matrix effect and carry-over were insignificant. Subsequently, the in vitro metabolism of PTU was assessed and UGT1A9 was identified as an important UGT isoform responsible for the glucuronidation of PTU. The information obtained from this study will facilitate future mechanistic investigation on the hepatoxicity of PTU and may optimize its clinical application.

## 1. Introduction

Propylthiouracil (PTU) is a thionamide medication commonly prescribed for the management of Graves’ disease and hyperthyroidism ever since the 1940s [1]. Besides inhibiting the synthesis of thyroid hormones by blocking the iodine oxidation in the thyroid gland, PTU also interrupts the conversion of tetraiodothyronine to triiodothyronine in peripheral tissues [2]. In comparison to methimazole [3,4], which is frequently used as a first-line therapeutic agent, PTU is preferred in the first trimester of pregnancy or treating thyroid storm/thyrotoxicosis [5]. Although PTU has good safety profiles in adult population with a relatively low incidence rate of hepatoxicity (1 in 10,000), an increased risk of acute liver failure was reported in the pediatric population [6,7,8]. Therefore, the U.S. Food and Drug Administration (FDA) issued a black box warning in 2009, followed by similar actions from the European Medicines Agency and the United Kingdom Medicines and Healthcare Regulatory Agency in the same year. So far, the exact mechanism of action of PTU-induced hepatotoxicity remains unclear.

PTU is commonly administered through the oral route and displays rapid absorption with an excellent bioavailability of 75% [9,10]. Plasma protein binding of PTU is as high as 80–85%. PTU is mainly eliminated by the liver through the formation of glucuronides or sulfates. Within 24 h, around 35% of the dosed PTU is excreted as metabolite forms in urine [11]. So far, the role of the proposed intermediates in hepatoxicity is unclear. Reactive metabolites produced by myeloperoxidase in neutrophils might be responsible for agranulocytosis; however, the presence of such intermediates has not been observed in the liver [12]. PTU or its metabolites might also affect some intracellular targets, which consequently mediate hepatoxicity. Moreover, it was reported that glutathione transferase and glutathione peroxidase were inhibited in a concentration-dependent manner by PTU and its sulfated metabolites [13].

Glucuronidation, which is catalyzed by uridine 5′-diphospho-glucuronosyltransferases (UGTs) is generally considered as an effective pathway for the elimination of xenobiotics. Similar to many well-known drugs including morphine, propofol, and acetaminophen, PTU is subjected to conjugation with propylthiouracil N-β-D glucuronide (PTU-GLU) as a metabolite [14,15]. Interestingly, UGT1A9/2B4 activities were found to be relatively low in infants (0.5–2 years) in comparison to adults [16,17,18,19]. This is probably due to lower transcription of UGT1A9/2B4, which had been observed in pediatric livers [18]. Insufficient UGTs activities may hinder the clearance of PTU, resulting in hepatotoxicity. Age-dependent activities of UGTs appear to be a reasonable explanation of the difference in safety profile between adults and infants [20,21,22]. Clearly, it is of scientific interest to identify the specific UGTs isoform that is responsible for the glucuronidation of PTU.

An accurate bioanalytical method for the quantification of PTU and PTU-GLU will enable further investigation on this topic. So far, several chromatographic methods including HPLC with ultraviolet (UV) [23], HPLC with Mass Spectrometry (MS) [24] and HPLC with iodine-azide reaction as detection systems [25] have been attempted. However, neither a validated analytical method for PTU-GLU nor a protocol for simultaneous quantification of PTU and PTU-GLU has been established.

In this study, a reliable HPLC-MS/MS method for the simultaneous quantification of propylthiouracil and its N-β-D glucuronide has been developed and validated. Glucuronidation of propylthiouracil has been subsequently examined in in vitro systems. The information obtained from this study will facilitate future mechanistic investigation on the hepatoxicity of PTU and may optimize its clinical application.

## 2. Results and Discussion

### 2.1. HPLC-MS/MS Conditions and Method Optimization

**Figure 1:**
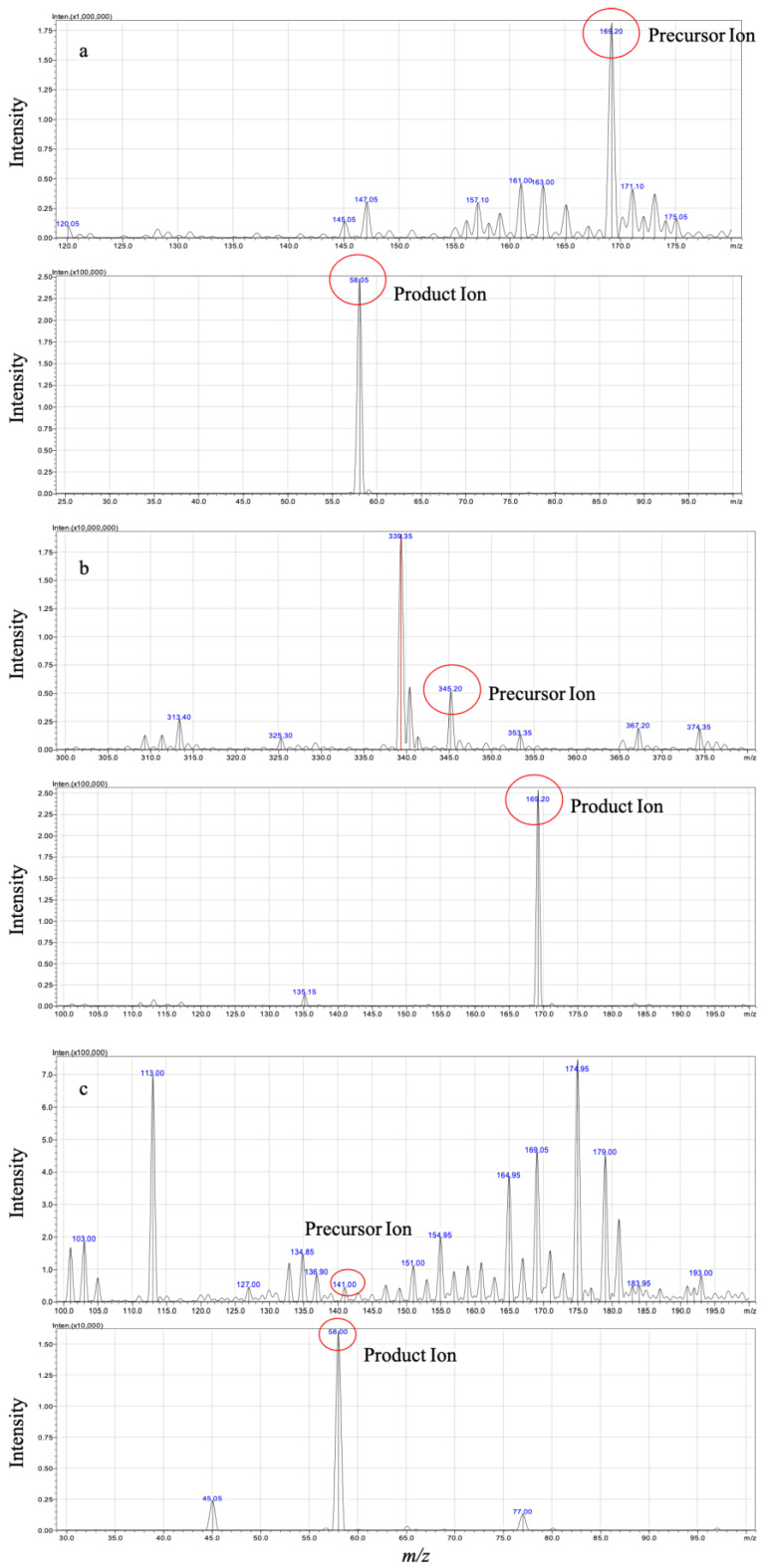
Mass spectra of PTU (**a**), PTU-GLU (**b**), and MTU (**c**) in ESI mode.

Through optimizing the instrumental parameters of mass spectrometry, we obtained sensitive and robust signals of the analytes. Methylthiouracil (MTU) is chosen as an internal standard (IS) due to its structural similarity to PTU. Full-scan and product ion scan were applied to identify the mass-spectra of PTU, its metabolite PTU-GLU and IS (Figure 1) in negative electrospray ionization (ESI) mode. During method optimization, it was observed that the strongest precursor ions > product ions signals were *m/z* 169.20 > 58.05, 345.2 > 169.20, and 141.0 > 58.00 for PTU, PTU-GLU, and MTU, respectively (shown in Figure 1).

**Figure 2:**
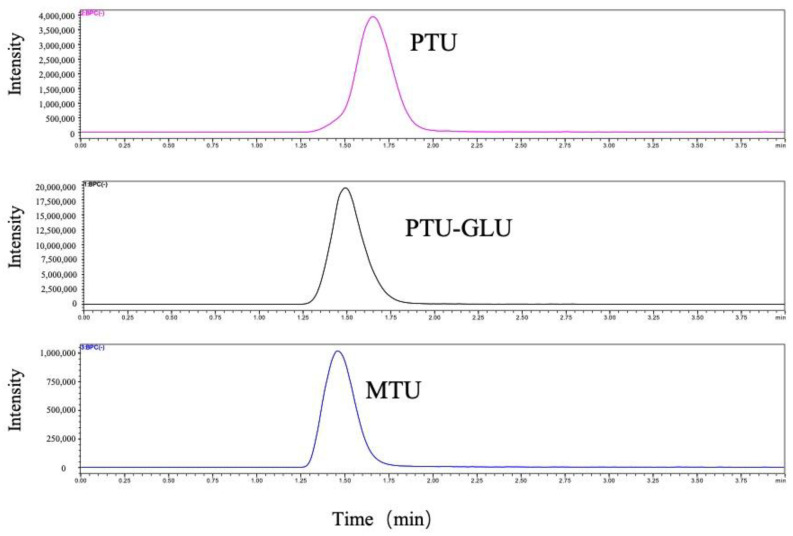
Representative MRM ion chromatograms of PTU, PTU-GLU and MTU.

Chromatographic separation of analytes was achieved on a ZORBAX Extend-C18 column (2.1 × 50 mm, 1.8 μm; Agilent Technologies, Waldbronn, Germany). PTU, PTU-GLU, and MTU eluted at 1.66 min, 1.50 min, 1.40 min, respectively with optimal peak shapes (Figure 2). The HPLC-MS/MS was controlled by the software of LabSolution and the experimental parameters were subsequently optimized while multiple reaction monitoring (MRM) was applied to measure the analytes.

**Figure 3:**
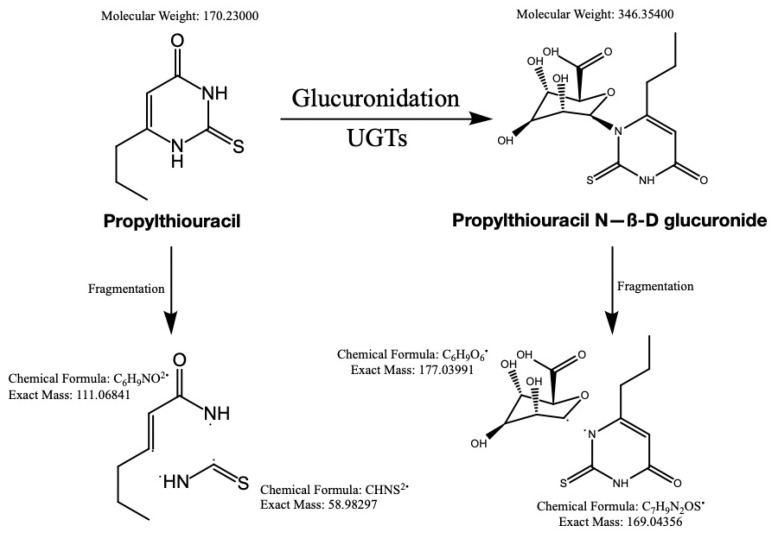
The structure of PTU and PTU-GLU, along with proposed fragmentation pathways.

The optimized HPLC-MS/MS parameters are listed in Table 1 and the proposed fragmentation pathway is illustrated in Figure 3.

### 2.2. Assay Validation

#### 2.2.1. Selectivity

**Figure 4:**
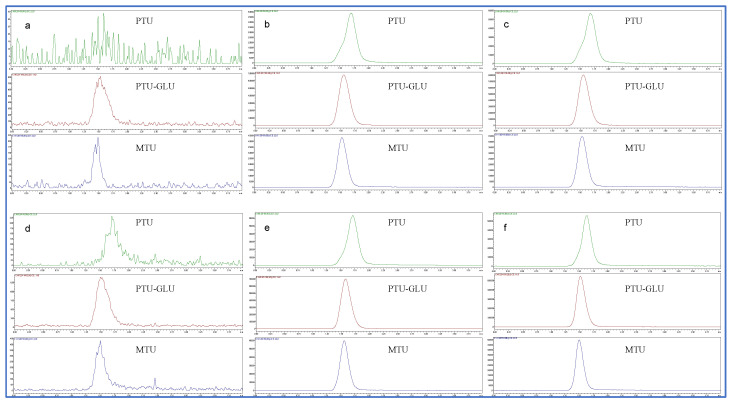
Representative chromatograms of HLMs and human recombinant UGT1A9 spiked with PTU (0.1 µM), PTU-GLU (0.1 µM) and MTU (IS, 10 µM). (**a**) blank HLMs; (**b**) inactive HLMs spiked with PTU, PTU-GLU and IS; (**c**) inactive HLMs spiked with PTU, PTU-GLU, IS and UGT1A9 inhibitor magnolol (50 µM); (**d**) blank human recombinant UGT1A9; (**e**) inactive human recombinant UGT1A9 with PTU, PTU-GLU and IS; (**f**) inactive human recombinant UGT1A9 spiked with PTU, PTU-GLU, IS and UGT1A9 inhibitor magnolol (50 µM).

This bioanalytical method displayed excellent selectivity. No notable interference for PTU, PTU-GLU and IS was observed at lower limit of quantification (LLOQ) (0.1 µM) in the presence of biological matrices such as human liver microsomes (HLMs) and human recombinant UGT1A9, and UGT inhibitor (magnolol). Representative chromatograms of HLMs/human recombinant UGT1A9 spiked with PTU, PTU-GLU and IS are shown in Figure 4.

#### 2.2.2. Sensitivity

The lower limit of quantitation (LLOQ) was established at 0.1 µM for both analytes with acceptable accuracies and precision (data not shown).

#### 2.2.3. Calibration Curve and Linearity

**Figure 5:**
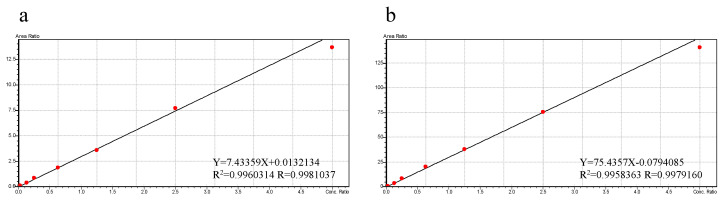
Representative calibration curves for PTU (**a**) and PTU-GLU (**b**).

As shown in Figure 5, the calibration curves with the optimal fit were established over the concentration ranging from 0.1 to 50 µM for PTU and PTU-GLU. Both *R*^2^ values of the calibration curves were higher than 0.99, indicating good linearity of the assay.

#### 2.2.4. Carry-Over

Absence of carry-over was confirmed as no signal interference was observed in blank samples following the highest calibrator (data not shown).

#### 2.2.5. Accuracy and Precision

The results of intra- and inter-day accuracy and precision are listed in Table 2. The experimental data fulfilled the guidelines’ requirements as the mean accuracies (%Bias) were all within 85–115% with %RSD values not more than 15% [26,27].

#### 2.2.6. Recovery and Matrix Effect

The recovery results for PTU and PTU-GLU, ranging from 98.2% to 114% with SD values within 15% are listed in Table 3. The matrix effects for both analytes were also found to be insignificant.

#### 2.2.7. Stability

The stability of the samples was evaluated by using six replicates of quality control samples at three concentration levels under different storage conditions, including benchtop, auto-sampler, long-term and freeze–thaw stability. The data are listed in Table 4. Again, the stability profiles fulfilled the guidelines’ requirement [26,27].

### 2.3. Application to In Vitro Metabolism Study

The analytical methods for the quantification of PTU in biological matrices such as plasma, milk, urine and tissue samples have been reported [24,28,29,30]. However, to our knowledge, there is no established protocol suitable for the examination of glucuronidation of PTU. Therefore, in the present study, we developed and validated a reliable method for the simultaneous measurement of PTU and PTU-Glu and subsequently applied it to study the in vitro metabolism of PTU in HLMs.

#### 2.3.1. Formation of PTU-GLU

**Figure 6:**
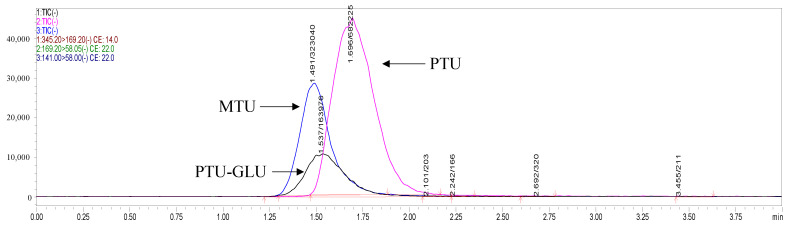
Representative HPLC profile of PTU and PTU-GLU. Propylthiouracil was incubated with the HLMs system at 37 °C for 60 min as described in Materials and Methods (Section 3.4).

**Figure 7:**
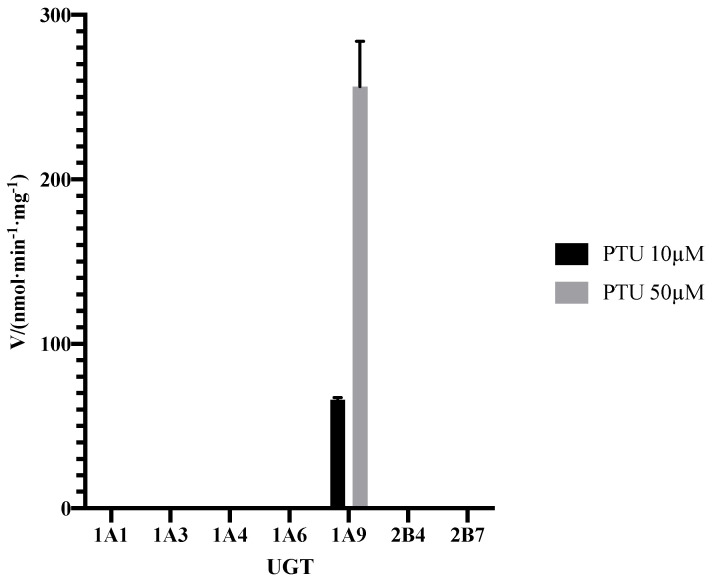
Formation rate of PTU-GLU by recombinant human UGTs. PTU was incubated with different UGTs at 37 °C for 60 min as described in Materials and Methods (Section 3.4). Each point represents the average of three replicates.

The formation of PTU glucuronide by HLMs was analyzed by the HPLC-MS/MS system, with the representative result illustrated in Figure 6. No metabolite was observed in the negative control. The result was consistent with the research published in 1977, which confirmed glucuronide conjugation of PTU in guinea pig liver microsome [15]. As shown in Figure 7, no PTU-GLU formation was observed in the incubation mixture in the absence of recombinant human UGT1A9 isoform. Clearly, UGT1A9 plays an important role in the glucuronidation of PTU.

#### 2.3.2. Inhibitory Study

**Figure 8:**
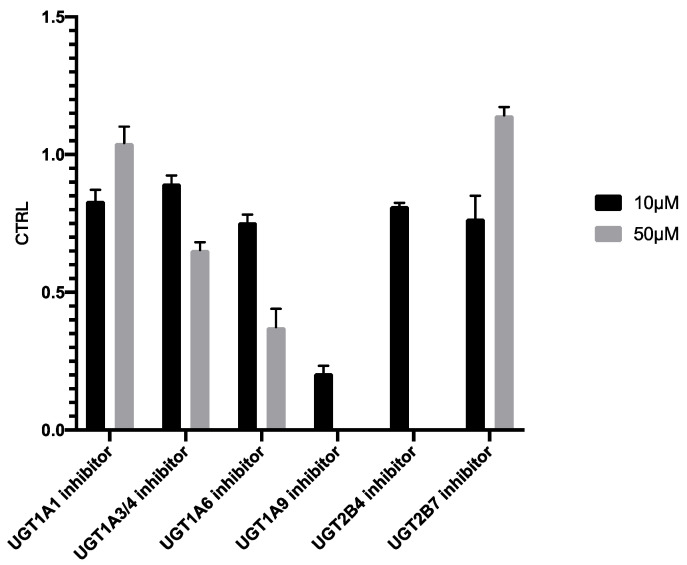
Inhibition of propylthiouracil glucuronidation in HLMs by different UGT inhibitors. Each point represents the average of three replicates.

**Figure 9:**
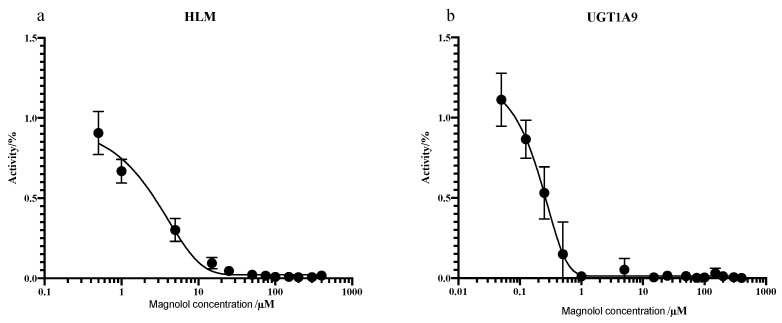
Inhibition of PTU glucuronidation in HLMs (**a**) and UGT1A9 (**b**) by magnolol. Each point represents the average of three replicates.

As shown in Figure 8, addition of 10 µM UGT1A9 inhibitor (magnolol) exhibited significant suppression on PTU glucuronidation, confirming our finding reported in Figure 7. Magnolol inhibited PTU glucuronidation in a concentration-dependent manner in both HLMs and UGT1A9 (Figure 9). Interestingly, the IC_50_ of magnolol for both HLMs and UGT1A9 were almost identical (Table 5), suggesting that UGT1A9 was the major UGT isoform in HLMs that mediates the glucuronidation of PTU. Of note, UGT2B4 inhibitor fluconazole showed a significant suppressive effect at a high concentration of 50 µM while other UGT inhibitors also slightly slowdown the glucuronidation. Such phenomenon may be due to their weak inhibitory specificity to UGT1A9. However, it is still unclear whether UGT1A9 is the only isoform of UGTs responsible for PTU glucuronidation.

#### 2.3.3. Enzymatic Kinetics Study

**Figure 10:**
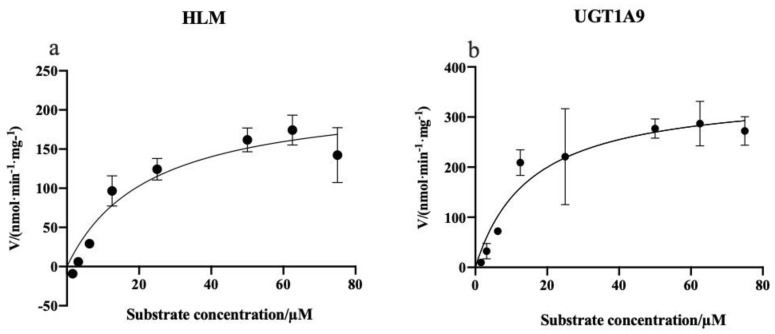
Kinetics of PTU glucuronidation by HLMs (**a**) and UGT1A9 (**b**). Each point represents the average of three replicates.

The PTU-GLU formation in the UGT1A9 and HLMs was analyzed to estimate the kinetic parameters. As illustrated in Figure 10, the substrate concentration-glucuronidation velocity curves exhibited typical Michaelis–Menten kinetics for both incubation systems. The mean *K_m_* value was calculated to be 15.27 µM for UGT1A9 and 22.76 µM for HLMs. For *V_max_*, the values were 352.3 nmol/min/mg and 220.0 nmol/min/mg, respectively (Table 6). Lower *K_m_* and higher *V_max_* indicate a higher affinity and catalytic activity. The results showed that PTU had a good affinity to UGT1A9. Based on the result of these in vitro experiments, a physiologically-based pharmacokinetic model of PTU could be developed and validated by clinical data, enabling an accurate prediction on its hepatotoxicity and the risk of drug-drug interaction [31,32,33,34].

## 3. Materials and Methods

### 3.1. Chemicals and Reagents

Propylthiouracil (PTU, purity > 98%) was obtained from Aladdin Biochemical Technology Co., Ltd. (Shanghai, China). Propylthiouracil N-B-D-glucuronide (PTU-GLU, purity > 98%) was provided by Shanghai ZZBIO Biochemical Technology Co., Ltd. (Shanghai, China). Methylthiouracil (MTU, internal standard, purity > 98%), hesperetin (purity > 98%), nilotinib (purity > 98%), magnolol (purity > 98%), silybin (purity > 98%), fluconazole (purity > 98%) and mefenamic acid (purity > 98%) were all purchased from Shanghai Yuanye Bio-Technology Co., Ltd. (Shanghai, China). Hecogenin (purity > 98%) was supplied from Chengdu Pufei De Biotech Co., Ltd. (Chengdu, China); uridine 5′-diphosphoglucuronic acid (UDPGA, purity > 98%) and alamethicin (purity > 98%) were obtained from Sigma-Aldrich Co., Ltd. (Merck, Shanghai, China). Pooled human liver microsomes (HLMs) and human recombinant UGT1A1 and 1A6 were purchased from Corning Incorporated (Corning, NY, USA). Other recombinant human UGT isoforms (1A3, 1A4, 1A9, 2B4 and 2B7) were obtained from Research Institute for Liver Diseases Co. Ltd. (Shanghai, China). Magnesium chloride (MgCl_2_, purity > 98%) and tris-(hydroxymethyl) aminomethane (Tris, purity > 98%) were purchased from Sinopharm Chemical Reagent Co., Ltd. (Shanghai, China). A Milli-Q-plus system (Millipore, Bedford, MA, USA) was utilized to generate ultrapure water. Other reagents were purchased from standard chemical suppliers and were of analytical grade or higher.

### 3.2. Instrumental Conditions of HPLC-MS/MS

Determination of the analytes was conducted with the HPLC-MS/MS-8060 system (Shimadzu Scientific Instruments, Columbia, MD, USA). The mobile phase A consisted of 0.1% of formic acid in water and phase B consisted of 0.1% formic acid in methanol and acetonitrile (2:1, *v/v*). Chromatographic separation was achieved on a ZORBAX Extend-C18 column (2.1 × 50 mm, 1.8 μm; Agilent Technologies, Waldbronn, Germany) through isocratic delivery of the mobile phase (A:B=40:60) at 0.1 L/min at 35°. The injection volume was 1 µL. Along with multiple reaction monitoring (MRM) mode, electrospray ionization was performed at its negative ion mode using nitrogen as the nebulizing, drying, and heating gas with the flow rate values set at 3.0, 10.0, and 10.0 mL/min, respectively.

The heat block temperature was set at 400 °C with the desolvation line (DL) temperature at 250 °C and interface temperature at 300 °C. Other operating parameters were set as follows: conversion voltage, 10 kV; detector voltage, 2.08 kV; collision induced dissociation (CID) gas, 270 kPa; interface voltage, 4.0 kV; nebulizer gas, 2.0 L/min; heating gas, 10 L/min; and drying gas, 10 L/min.

### 3.3. Calibration Standards and Quality Control (QC) Samples

PTU primary stock solution was prepared by dissolving 8.5 mg of PTU in 5 mL of methanol: water (1:1, *v/v*) to obtain a final concentration of 1.70 mg/mL (10 mM). To make a stock solution of 1 mg/mL PTU-GLU (2.86 mM), 1 mg of PTU-GLU was dissolved into an appropriate amount of methanol. For IS, the stock solution of MTU (1 mM) was prepared by dissolving 3.6 mg of MTU in 25 mL of methanol: acetonitrile (2:1, *v/v*). All stock solutions were transferred to Eppendorf vials and stored at −20 °C before usage.

The calibration standards and quality control samples were prepared by proper dilution of the stock solutions with Tris-HCl solution and mixed with inactivated HLMs, alamethicin, and MgCl_2_. The concentrations of calibration standards ranged from 0.1 µM to 50 µM (0.1, 0.5, 1, 5, 10, 25 and 50 µM) for PTU and PTU-GLU. At the same time, QC concentrations were set at 0.1 µM (LLOQ), 0.5 µM (quality control at low concentration), 5 µM (quality control at middle concentration), and 25 µM (quality control at high concentration). The final IS concentration was 10 µM. Samples used for calibration and QC were freshly prepared at the beginning of the experimental day.

### 3.4. Biological Sample Preparation

The in vitro metabolic study was carried out using a protocol modified from a previous report [35]. To formulate the incubation system, a 10 µL mixture of alamethicin (250 µg/mL) and MgCl_2_ (50 mM), precalculated amounts of PTU (200 µM) and Tris-HCl (50 mM, pH = 7.4) were mixed vigorously and kept on ice for 15 min. Upon addition of 5 µL of HLMs (5 mg/mL) or recombinant human UGTs (2 mg/mL), preincubation was applied at 37 °C for 10 min, followed by the addition of 10 µL of UDPGA (25 mM) to start the reaction. Then, 60 min later, a double volume of ice-cold MTU acetonitrile solution (10 µM) was added into the system along with thorough vortexing for termination. Centrifugation at 18,000g was then performed at 4 °C for 15 min to obtain supernatant for HPLC-MS/MS analysis.

### 3.5. Assay Validation

This bioanalytical method was validated in accordance with the guidelines from the FDA and International Conference on Harmonization of Technical Requirements for Registration of Pharmaceuticals for Human Use (ICH) guidelines [26,27].

The confirmation of selectivity was achieved by evaluating the possible interference from the biological matrix and inhibitor. The acceptance criteria are defined as no co-eluting peaks greater than 20% of the PTU/PTU-GLU at the LLOQ level and 5% for IS.

The sensitivity is represented by LLOQ and it is identified as the guidelines specified [26,27].

Nine calibration standards, including seven non-zero and two blank groups (one with IS and the other without), were used to construct the calibration curves by plotting the peak area ratios (analyte/IS) vs. concentrations of the analytes. Linear regression (y = mx + c) and weighted (1/x^2^) values were utilized to produce the calibration curve with correlation coefficients (R^2^) greater than 0.99.

The carry-over was assessed by analyzing two blank samples following the most concentrated calibrator. With the peak area less than 20% of LLOQ and 5% of IS, the carry-over effect could be considered negligible.

Inter-day accuracy and precision were determined by analyzing samples at each quality control concentration daily for three successive days, while intra-day values were evaluated by the analysis of the quality control samples on the same day. All analyses were performed in six replicates. Bias in terms of percentage was utilized and expected to be within ±15% (except for ±20% at LLOQ) for good accuracy. Precision was defined as the %RSD with the same acceptance criteria.

To assess the matrix effect, blank HLMs were spiked with IS, PTU, and PTU-GLU at low, middle and high quality control concentrations. Neat solutions for both analytes were also prepared with methanol at the same concentration levels. The IS normalized matrix factor was used to determine the matrix effect using Equation (1) shown below.

(1) $$\begin{array}{c} \mathrm{IS}\;\mathrm{Normalized}\;\mathrm{Matrix}\;\mathrm{Effect}\\ \;\;\;\;\;\;\;\;\;\;\;\;\;\;\;\;\;\;\;\;\;\;\;\;\;\;\;\;\;\;=\frac{Mean\;peak\;area\;ratio\;of\;analyte/IS\;in\;matrix}{Mean\;peak\;area\;ratio\;of\;analyte/IS\;in\;solvent\;}\times 100\% \end{array}$$

Analytes at low, middle and high quality control concentrations and IS were incubated in HLMs as described above (Section 3.4), followed by centrifugation. Supernatant was obtained for chromatographic analysis (Signal I). Similarly, analytes free supernatant was first recovered from the HLMs and then mixed with analytes and IS at the same level (Signal II). The recovery was determined by comparing the peak areas using the following Equation (2).

(2) $$\mathrm{Recovery}\;\left(\%\right)=\frac{Analyte\;Signal\;I}{Analyte\;Signal\;II}\times 100\%$$

Similar calculations can be found in a previous report [36].

Benchtop (short-term) stability was conducted under the common laboratory conditions (20 °C) for 6 h (the same duration of the experiments). The stability of analytes in the matrix stored in the −80 °C freezer was analyzed to evaluate long-term variation. To assess the impact of repeatedly removing samples from the freezer, the freeze-thaw stability of the analytes was performed after three freeze-thaw cycles. Similarly, auto-sampler stability was also carried out. A mean percentage of analyte remaining ranging from 85% to 115% indicates sample stability.

### 3.6. In Vitro Metabolism Study

#### 3.6.1. Assays of PTU-GLU Determination

The formation of PTU-GLU was attempted in reaction systems containing recombinant human UGT1A1, 1A3, 1A4, 1A6, 1A9, 2B4, 2B7 or pooled HLMs, using the incubation method described above for the in vitro study and HPLC-MS/MS analysis.

#### 3.6.2. Enzymatic Inhibition Study

Several small-molecule chemicals have demonstrated potent and selective inhibitory effects towards different UGT isoforms, which could be utilized for further screening. In the initial study, HLMs incubation systems were mixed with nilotinib (UGT1A1 inhibitor) [37], hecogenin (UGT1A3/1A4 inhibitor) [38], silybin (UGT1A6 inhibitor) [39], magnolol (UGT1A9 inhibitor) [30], fluconazole (UGT2B4 inhibitor) [40] or mefenamic acid (UGT2B7 inhibitor) [41] at two different concentrations, namely 10 µM and 50 µM. Based on the results from the initial study, magnolol was further tested for its inhibitory effect on PTU glucuronidation in HLMs and UGT1A9. Various concentrations of magnolol (0.1–400 µM) were added to the incubation system to determine the half-inhibition concentrations (IC_50_) as described in Section 3.4.

#### 3.6.3. Kinetic Study

To evaluate the kinetic parameters for glucuronidation, PTU at various concentrations (1–75 μM) was incubated with HLMs or recombinant human UGT1A9 separately.

Enzymatic kinetics of metabolism follows a simple Michaelis–Menten equation that describes the relationship between substrate concentration and reaction velocity (Equation (3)).

(3) $$V=\frac{V_{max}\times \left[S\right]}{K_{m}+\left[S\right]}$$

where *V* is defined as the initial velocity of the metabolic reaction and *V_max_* is the maximum rate. [S] stands for the concentration of substrate and *K_m_* is the Michaelis constant defined as the substrate concentration at half of the *V_max_*.

## 4. Conclusions

The HPLC-MS/MS method for the simultaneous quantification of PTU and its metabolite (PTU-GLU) was successfully developed and validated. This reliable bioanalytical protocol was subsequently applied to examine the in vitro metabolism of PTU. Glucuronidation of PTU was confirmed in pooled human HLMs while UGT1A9 was identified as an important UGT isoform responsible for the glucuronidation of PTU. The information obtained from this study will facilitate future mechanistic investigation on the hepatoxicity of PTU and may optimize its clinical application. Further investigation on this topic is warranted.

## Figures and Tables

**Table 1:** Summary of HPLC-MS/MS parameters.

	PTU	PTU-GLU	MTU(IS)
MRM Transition *m/z* (Q1-Q3)	169.2 > 58.05	345.2 > 169.2	141.00 > 58.00
MS Ionization	ESI mode		
Q1 (V)	12.0	13.0	10.0
Q2 (V)	22.0	14.0	22.0
Q3 (V)	20.0	17.0	19.0
Column	ZORBAX Extend-C18 column (2.1 × 50 mm, 1.8 μm)		
Column Temperature	35 °C		
Run Time	4 min		
Mobile Phase	Phase A: water (0.1% formic acid) Phase B: methanol/acetonitrile (0.1% formic acid) (2:1, *v/v*)		
Flow	0.1 mL/min (40:60 = A:B)		
Injection Volume	1 µL		
Retention Time	1.66 min	1.50 min	1.40 min

**Table 2:** Percentage bias and relative standard deviation results of intra- and inter-day assay for PTU and PTU-GLU.

		PTU				PTU-GLU			
		0.1 µM	0.5 µM	5 µM	25 µM	0.1 µM	0.5 µM	5 µM	25 µM
Intra-Day Assay (*n* = 6)	Measured ^a^	0.107 ± 0.0054	0.574 ± 0.038	5.60 ± 0.62	24.9 ± 0.5	0.0907 ± 0.0085	0.575 ± 0.018	5.50 ± 0.30	26.3 ± 0.9
	%Bias	7.58%	14.8%	11.3%	−0.485%	−9.33%	14.9%	10.0%	5.05%
	%RSD	5.02%	6.61%	9.42%	9.87%	9.38%	7.33%	5.42%	3.35%
Inter-Day Assay (*n* = 6)	Measured ^a^	0.106 ± 0.0079	0.526 ± 0.51	5.40 ± 0.73	24.9 ± 2.5	0.0890 ± 0.0085	0.547 ± 0.040	5.74 ± 0.35	28.0 ± 1.0
	%Bias	6.35%	5.21%	8.02%	−0.225%	−11.0%	9.47%	14.7%	11.9%
	%RSD	7.44%	5.46%	11.2%	2.12%	6.26%	3.19%	6.12%	3.65%

^a^ Measured mean concentration (µM, *n* = 6) ± SD.

**Table 3:** Recovery and matrix effect of PTU and PTU-GLU from human liver microsomes.

		PTU			PTU-GLU		
		0.5 µM	5 µM	25 µM	0.5 µM	5 µM	25 µM
Recovery (*n* = 6)	Measured ^a^	101.7% ± 4.2%	115.1% ± 14.0%	114.2% ± 1.7%	114.6% ± 12.1%	98.2% ± 8.6%	106.1% ± 0.6%
	%RSD	4.1%	12.2%	1.5%	10.6%	8.8%	0.6%
Matrix Effect (*n* = 6)	Measured ^b^	100% ± 9.7%	86.0% ± 11.8%	100% ± 12.2%	99.3 ± 8.6%	109% ± 6.9%	87.6% ± 4.1%
	%RSD	9.7%	12.9%	12.2%	8.7%	6.3%	4.7%

^a^ Mean recovery calculated by Equation (2) (*n* = 3) ± SD. ^b^ Mean matrix effect calculated by Equation (1) (*n* = 3) ± SD

**Table 4:** Stability results for PTU and PTU-GLU at different storage conditions.

Storage Conditions		PTU			PTU-GLU		
		0.5 µM	5 µM	25 µM	0.5 µM	5 µM	25 µM
Benchtop (*n* = 6) 20 °C, 6 h	Measured ^a^	0.428 ± 0.011	5.13 ± 0.15	24.9 ± 2.7	0.558 ± 0.035	5.63 ± 0.54	25.47 ± 1.2
	%Bias	−14.5%	2.69%	0.473%	11.8%	12.6%	1.87%
	%RSD	2.58%	2.83%	11.0%	6.22%	9.62%	2.34%
Auto-Sampler (*n* = 6) 4 °C, 72 h	Measured ^a^	0.496 ± 0.030	5.54 ± 0.16	24.8 ± 1.0	0.49 ± 0.039	4.47 ± 0.32	23.5 ± 0.9
	%Bias	−7.67%	10.8%	0.885%	−12%	−10.5%	−6.06%
	%RSD	5.97%	2.91%	0.416%	7.86%	7.36%	3.91%
Long-Term (*n* = 6) −80 °C, 20 d	Measured ^a^	0.481 ± 0.029	4.95 ± 0.46	23.8 ± 2.6	0.429 ± 0.45	5.88 ± 0.82	22.7 ± 3.0
	%Bias	−3.97%	−0.893%	−4.76%	−14.4%	7.53%	−8.78%
	%RSD	6.06%	9.28%	10.9%	10.5%	14.5%	13.2%
Freeze–Thaw (*n* = 6) −80 °C, Up to 3 Cycles	Measured ^a^	0.455 ± 0.056	5.41 ± 0.68	26.3 ± 1.0	0.517 ± 0.067	5.20 ± 0.42	25.7 ± 2.6
	%Bias	−9.04%	−8.19%	5.09%	3.43%	4.01%	2.66%
	%RSD	12.3%	12.6%	3.96%	13.0%	8.15%	10.0%

^a^ Mean recovery calculated by Equation (2) (*n* = 3) ± standard deviation (SD).

**Table 5:** Inhibition of PTU glucuronidation in HLMs and recombinant UGT1A9 by magnolol.

	Magnolol IC_50_ (µM)
HLMs	1.028
UGT1A9	1.160

**Table 6:** Kinetic parameters of propylthiouracil glucuronidation in HLMs and recombinant human UGT1A9.

Enzyme	*K_m_* (µM) ^a^	*V_max_* (nmol/min/mg) ^a^	Clearance (mL/min/mg) ^b^
HLMs	22.76 ± 12.29	220.0 ± 43.35	9.67
UGT1A9	15.27 ± 7.73	352.3 ± 56.38	23.07

^a^*K_m_* and *V_max_* were presented as mean value ± SD (*n* = 6). ^b^ The clearance values were estimated by *V_max_*/*K_m_*.

## Data Availability

Data are contained within the article.

## Abbreviations

ESI, electrospray ionization; HLMs, human liver microsomes; IS, internal standard; LLOQ, lower limit of quantiifcation; MRM, multiple reaction monitoring; MS, mass spectrometry; MTU, methylthiouracil; PTU, propylthiouracil; PTU-GLU, propylthiouracil glucuronide; UGTs, uridine 5′-diphospho-glucuronosyltransferases.
